# Soil mineralogy and potassium forms distribution and release in some calcareous soils of the Darab Region, Iran

**DOI:** 10.1371/journal.pone.0334155

**Published:** 2025-12-19

**Authors:** Mahnaz Javan, Ali Abtahi, Gholamreza Zareian, Hasan Haghighatnia

**Affiliations:** 1 Department of Soil Science, Marv.C., Islamic Azad University, Marvdasht, Iran; 2 Soil and Water Research Department, Fars Agricultural and Natural Resources Research and Education Center, AREEO, Shiraz, Iran; ICAR National Bureau of Soil Survey & Land Use Planning, INDIA

## Abstract

Understanding the distribution and release dynamics of potassium (K) in calcareous soils is essential for efficient nutrient management in arid and semi-arid regions. This study investigates K forms and their release behavior in ten agricultural soil pedons from the Darab region in southern Iran, characterized by diverse physiographic settings and soil orders (Entisols, Inceptisols, Aridisols, and Vertisols). Dominant clay minerals identified through X-ray diffraction included illite, smectite, and chlorite. The surface horizons showed greater average concentrations of soluble K (0.63%), exchangeable K (5.06%), and non-exchangeable K (6.5%), whereas structural K was most abundant in subsurface horizons (89.32%). Cumulative K release, assessed through successive CaCl₂ extractions, ranged from 267.7 to 572.4 mg/kg in surface soils and 145.2 to 433 mg/kg in subsurface layers. Release kinetics of non-exchangeable K were effectively described by the power function and pseudo-second-order models, indicating both rapid and sustained release phases. Among the studied soils, pedon 6 (Inceptisol) exhibited the highest release rate, as reflected in its b parameter suggesting enhanced K availability and faster equilibrium attainment. These findings underscore the critical influence of soil mineralogy on potassium dynamics and support the development of site-specific fertilization strategies.

## 1. Introduction

Potassium (K) is recognized as an essential nutrient for the growth and metabolism of plants, animals, and humans [1–4]. Although K accounts for approximately 2.6% of the Earth’s crust [5,6], it predominantly exists in mineral forms within the soil, particularly in clay minerals such as muscovite, biotite, illite, and feldspar [7–10]. If not adequately replenished through fertilization, especially under intensive cropping systems, soil K reserves gradually diminish, resulting in reduced plant performance and lower agricultural productivity [11].

In recent decades, the depletion of soil K has emerged as a major challenge in highly cultivated regions [12]. The quantity and bioavailability of K are strongly influenced by the physical and chemical characteristics of soils, including mineral composition, clay type and content, organic matter, and climatic variables such as temperature and moisture regimes [13]. These factors cause substantial variation in K status among soils. Thus, identifying and quantifying the different forms of K is critical to understanding its availability and guiding nutrient management strategies.

**Fig 1:**
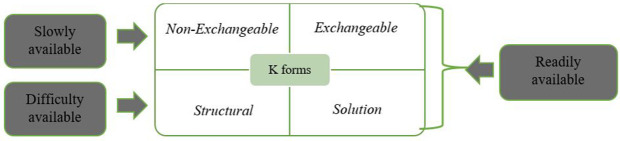
Classification of different forms of K in soil (Panda et al., 2022).

Soil K is commonly categorized into four distinct forms: soluble, exchangeable, non-exchangeable, and structural [1,14–16]. While plants primarily absorb potassium from the soluble and exchangeable pools present in the soil solution and on colloidal surfaces [9], these forms represent only a small fraction of the total potassium content. The vast majority of soil K—typically ranging between 90% and 98%—is retained in non-exchangeable and structural forms [17]. As depicted in Fig 1, the relative contribution of each form varies depending on soil type and mineralogical composition.

Several factors influence the transformation and distribution of K among these forms, including clay mineralogy, texture, organic matter, soil water status, and temperature fluctuations [12,13,15,18,19]. In particular, soils with higher proportions of 2:1 clay minerals (e.g., illite and smectite) are more likely to retain and slowly release K over time. In the calcareous soils of Iran—especially those under intensive agriculture—exchangeable K may not reliably represent plant-available K [20,19]. This is due to complex interactions between soil constituents and the limited mobility of K ions in high-pH conditions [21,22]. Therefore, evaluating K release from non-exchangeable forms becomes essential, particularly when assessing subsoil fertility across depth profiles [23].

Several studies have highlighted the contribution of non-exchangeable K as a medium-term K source under K-depleted or fertilized conditions [21,24]. However, limited research has been conducted on deeper soil layers, despite evidence suggesting that plant roots access K from multiple depths [17]. Moreover, the relationship between the type and quantity of clay minerals and K availability has garnered increasing interest in Iran and other arid to semi-arid regions [16,17,25–27]. One of the persistent challenges in these environments is the progressive reduction of soil K in the absence of targeted K fertilization [11,28].

Given the above context—and the scarcity of comprehensive studies focusing on K dynamics in calcareous soils of the Darab region in Fars Province, southern Iran—this study was undertaken with two primary objectives: **(i)** to assess the distribution of different forms of potassium across selected agricultural soils in relation to their mineralogical composition and degree of soil development, and

**(ii)** to investigate the kinetics of potassium release across soil horizons, with emphasis on the role of mineralogical properties.

## 2. Materials and methods

### 2.1. Study area

This study was conducted in the Darab region of Fars Province, southern Iran, which is one of the study areas investigated by the Soil and Water Research Department, Agricultural and Natural Resources Research Center, AREEO, IR Iran. It should be noted that the third author is academic staff of AREEO and no permission was required to access the field site. According to a previous study, ten soil profiles were selected in Darab regions, southern Iran with different physiography and the same agricultural capabilities. Soil profiles were dug, described, and classified according to Keys to Soil Taxonomy [29]. In the sampling points, information such as landform unit, soil parent material, slope, erosion status, drainage status, and land use were recorded. Then diagnostic horizons were distinguished and soil samples (a total of 38) were taken and transferred to the laboratory for soil analysis after air-drying and sieving (<2 mm).

### 2.2. Physicochemical properties of soils

Soil particle-size distribution was determined using the hydrometer method [30]. Soil pH was measured using the saturated paste extract, and electrical conductivity (EC) was measured in soil saturation extract using a conductometer. Organic carbon (OC) was determined by the wet oxidation method [31]. Cation exchangeable capacity (CEC) was measured by replacing exchangeable cations by NaOAc and exchanging Na^+^ with NH_4_OAc [32].

### 2.3. Mineralogical analysis

Before mineralogical analysis, samples were washed with distilled water to remove soluble salts and gypsum. Then carbonates, organic materials, and iron oxides were removed using 1 N Na acetate (pH 5), 30% H_2_O_2_, and dithionite citrate bicarbonate solution, respectively [33–35]. After the separation of clay fraction, samples were saturated with Mg and K, using 1 N MgCl_2_ and 1 N KCl, respectively. The Mg and K saturated samples were saturated by ethylene glycol and heated at 550^°^C, respectively. In addition, samples were treated with 1 N HCl to discriminate kaolinite and Fe-chlorite. These five treatments (Mg saturated, K saturated, Mg ethylene glycol (Mg-EG), K-550^°^C, and HCl treatments) were analyzed by XRD (X-ray diffraction). The relative abundance of clay minerals based on peak intensities was semi-quantitatively measured following the method of Johns et al. [36].

### 2.4. K forms

Different forms of K were determined for all 38 samples. Soluble K in the saturated extract was measured. Exchangeable K was determined through extraction with 1M NH_4_OAC at pH = 7 for 5 min [37] and non-exchangeable K with boiling 1 M HNO_3_ for 1 hour [38]. Total K was determined following digestion (383 K) of a 0.5-g soil sample with 10 mL of 48% HF and 1 mL of aqua regia (one part of concentrated HNO3 with three parts of concentrated HCl). Then structural K was calculated by subtracting total K from K extracted with boiling HNO_3_. K was measured on all filtered extracts using a Corning 405 flame photometer [39]. All analyses were carried out in triplicate and the mean values are presented.

### 2.5. Kinetic of K release

After preliminary analysis, 20 surface and subsurface soil samples were selected for the K release experiment. Kinetics of non-exchangeable K release was studied by successive extraction of 2g soil sample with 0.01 M CaCl_2_ [40–42] during 2, 4, 8, 24, 48, 72, 96, 120, 144, 168, 192, 360, 528, 696, 864, 1032 and 1200 minutes in two replications. The concentration of K in the supernatant was measured by a flame photometer (Corning 405). The release of non-exchangeable K with time was fitted to the following equations:

Power function equation: lny=lna+blnt

Elovich equation: y=a+blnt

Parabolic diffusion equation: $y=a+bt^{\text{1}/\text{2}}$

Psuedo second order $\frac{t}{y}=\frac{\text{1}}{qe}(t)+\frac{\text{1}}{k(qehat\text{2}}$

Where *y* was the quantity of K released (mg/kg) at time *t*, and (*qe, k, a*, *b)* was constants [17,43]. An essential concept of these equations is the invariant b, which is representative of the release rate of K. These mathematical models were evaluated through least squares regression analysis to ascertain which equation most accurately represented K release from soils. Coefficients of determination (r^2^) were derived from least squares regression of observed versus anticipated values. The rate constants of K release from soils were computed based on these models.

### 2.6. Data analysis

Statistical analysis of data was conducted utilizing the SPSS 20.0 program (SPSS Inc., Chicago, IL, USA) and all statistical tests were considered significant at p < 0.05. The kurtosis and skewness were ascertained for the evaluation of the data normality. Tukey HSD post hoc test was employed to ascertain the significance of variables among different groups. All graphs were generated in Microsoft Office Excel 2013.

## 3. Results

### 3.1. Soil classification

Based on soil temperature and moisture regimes, along with horizon development, the studied profiles were classified into four soil orders: Entisols (pedons 1, 2, 3, 5, 8, and 10), Inceptisols (pedons 4 and 6), Aridisols (pedon 9), and Vertisols (pedon 7). These soils formed under varying physiographic conditions, including gravelly colluvial fans, piedmont alluvial plains, and lowland settings. Given the limited rainfall in Fars Province, soil development is often minimal, and soil characteristics closely reflect those of the parent material. Selected physical and chemical properties of the soils are provided in Table 1. Clay content ranged from 15.9% to 44.2%, both extremes observed in Entisols (pedons 2 and 8). Cation exchange capacity (CEC) varied from 11.2 Cmol(+)/kg (Entisols, pedon 10) to 25.8 Cmol(+)/kg (Aridisols, pedon 9). The highest organic carbon content was recorded in the subsurface of pedon 5 (Entisols), while the greatest electrical conductivity (EC) was observed in pedon 9 (Aridisols). Soil pH values ranged from 7.4 to 8.2, with the highest in pedon 5.

**Table 1:** General characteristics and physico-chemical properties.

Pedon	Horizon	Physiographic Unit	Horizon	Classification USDA	TSS	pH	EC	OC	CEC	Texture Class	Sand (%)	Clay (%)
1	1	Gravelly Colluvial Fan	A	Aridic Ustorthents	50.10	7.8	2.02	0.90	14.10	Loamy- skeletal	47.00	20.80
	2		C_1_		60.00	8	0.75	0.23	11.20		54.10	18.00
2	1	Piedmont Alluvial Plains	Ap	Aridic Ustorthents	47.00	7.6	2.80	0.91	21.20	Fine	18.00	38.20
	2		C_1_		49.20	7.9	4.20	0.62	19.20		23.20	35.10
	3		C_2_		43.80	8.0	7.51	0.33	30.50		14.40	55.00
	4		C_3_		44.80	7.8	7.42	0.21	24.20		20.00	44.20
3	1	Piedmont Alluvial Plains	Ap	Aridic Ustorthents	45.00	7.8	3.28	0.70	17.10	Fine- Loamy	16.00	28.00
	2		C_1_		45.50	7.9	3.33	0.44	19.00		19.60	35.00
	3		C_2_		47.00	7.9	2.80	0.29	16.40		27.00	29.70
	4		C_3_		48.50	7.8	4.20	0.16	15.00		27.00	26.00
4	1	Piedmont Alluvial Plains	Ap	Aridic Haplustepts	52.50	7.8	1.90	0.80	22.40	Fine	22.00	34.00
	2		Bw_1_		53.50	8.1	0.43	0.50	20.00		22.60	36.00
	3		Bw_2_		58.50	8.1	0.48	0.40	21.80		15.00	40.00
	4		Bw_3_		61.50	8.2	1.20	0.18	20.80		17.10	39.70
5	1	Gravelly River Alluvial	A	Aridic Ustifluvents	46.00	8.0	13.6	0.85	24.80	Fine	18.20	38.60
	2		C_1_		39.00	8.2	17.7	0.22	26.40		23.60	35.00
	3		C_2_		39.00	8.2	25.4	0.19	15.10		25.60	24.00
	4		C_3_		42.00	7.7	22.4	0.14	20.30		20.10	33.00
6	1	Piedmont Alluvial Plains	Ap	Aridic Calciustepts	47.00	7.8	3.10	0.48	14.00	Fine- Loamy	23.80	25.20
	2		Bw		50.70	7.9	6.20	0.33	12.50		19.90	22.10
	3		Bk_1_		48.20	8.0	5.60	0.25	16.00		11.80	29.40
	4		Bk_2_		48.70	8.0	3.72	0.18	16.40		11.80	30.50
7	1	Piedmont Alluvial Plains	Ap	Aridic Calciustert	38.00	7.4	5.13	0.50	22.00	Fine	18.00	40.60
	2		Bss		37.00	7.6	6.10	0.40	25.10		26.00	38.80
	3		Bkss_1_		70.50	7.7	6.20	0.36	23.30		45.40	34.40
	4		Bkss_2_		71.50	7.8	6.25	0.18	18.40		42.20	33.40
8	1	Piedmont Alluvial Plains	A	Torrertic Ustorthents	48.90	7.8	4.18	0.90	12.50	Fine	37.20	15.90
	2		C_1_		50.50	7.9	12.1	0.25	12.60		30.60	22.60
	3		C_2_		50.50	8.0	13.3	0.16	14.00		25.60	22.60
	4		C_3_		45.00	7.8	18.1	0.18	15.00		23.40	24.40
9	1	Low Land	A	Typic Haplosalids	44.00	7.8	23.1	0.50	25.80	Fine	17.20	39.60
	2		Bz		39.00	8.1	17.1	0.22	24.40		21.60	37.00
	3		C_1_		35.00	8.1	24.3	0.18	16.10		25.20	24.40
	4		C_2_		39.00	7.7	33.1	0.13	20.40		19.80	33.40
10	1	Gravelly River Alluvial	Ap	Aridic Ustifluvents	51.50	7.8	1.90	0.60	13.20	Loamy- skeletal	33.20	21.60
	2		C_1_		52.50	7.9	0.46	0.22	9.20		45.20	16.00
	3		C_2_		50.00	7.9	0.37	0.90	22.80		37.20	19.60
	4		C_3_		55.00	8.0	0.39	0.30	18.80		34.20	17.20

EC: electrical conductivity (dS/m); OC: organic carbon (%); CEC: cation exchange capacity (Cmol(+)/kg).

### 3.2. K forms

**Fig 2:**
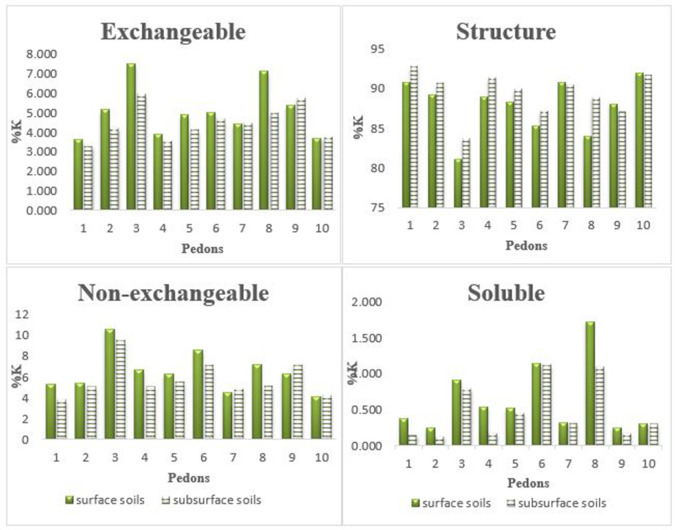
Potassium percentage in four pools in study topsoil (1) and subsoils (2).

As shown in Fig 2, the distribution of K forms varied significantly across pedons and horizons. The structural K fraction was highest in pedon 9 (Aridisols) at 4791 mg/kg, whereas the lowest soluble K value (1.65 mg/kg) was found in pedon 10 (Entisols), both in subsurface horizons. The maximum values for soluble, exchangeable, non-exchangeable, and structural K across all profiles were 42.4, 289, 357, and 4791 mg/kg, respectively. Pedon 9, with high CEC (21.7 Cmol^(+)^/kg) and clay content (33.6%), contained the highest exchangeable, non-exchangeable, and structural K concentrations. Pedon 8, with moderate CEC (13.5 Cmol^(+)^/kg) and 21.4% clay, exhibited the greatest soluble K. Statistical analysis revealed significant positive correlations between CEC and structural K (r = 0.640, p < 0.01), non-exchangeable K (r = 0.511, p < 0.01), and exchangeable K (r = 0.552, p < 0.01) (Table 2). In contrast, soluble K was negatively correlated with CEC (r = –0.349, p < 0.05). Similarly, positive correlations were found between clay content and exchangeable K (r = 0.511), non-exchangeable K (r = 0.540), and structural K (r = 0.630), while sand content was negatively associated with exchangeable K (r = –0.322) and non-exchangeable K (r = –0.496). EC also correlated positively with exchangeable K (r = 0.353, p < 0.05), whereas no significant correlation was found for OC and pH with any K form. Fig 2 illustrates K form distribution in surface and subsurface soils. Pedon 8 had the highest percentage of soluble K in surface soil, while pedon 6 showed the highest in subsurface soil. Pedon 3 exhibited the greatest exchangeable and non-exchangeable K percentages in both horizons, likely due to favorable temperature–moisture regimes and dominant illite mineralogy [12,44]. Non-exchangeable K was calculated by subtracting exchangeable K (extracted with 1M NH₄OAc) from K released by boiling HNO₃. It differs from structural K, as it resides in interlayer positions of 2:1 clay minerals such as vermiculite and illite and can be gradually mobilized under depletion conditions. In most pedons, the percentage of exchangeable and non-exchangeable K was higher in surface soils than in subsurface layers, except for pedons 7, 9, and 10. Structural K was generally more abundant in subsoils, with exceptions again being pedons 7, 9, and 10. These patterns suggest enhanced mineral weathering in surface soils, facilitated by more favorable temperature and moisture conditions.

**Table 2:** Pearson correlations coefficient of some soil characteristics and potassium forms.

Pearson Correlations											
	TSS	pH	EC	OC	CEC	Sand	Clay	Soluble	Exchangeable	Non-exchangeable	Structural
TSS	1										
pH	0.081	1									
	0.63										
EC	−.543**	0.079	1								
	0	0.638									
OC	0.019	−0.303	−.398*	1							
	0.909	0.064	0.013								
CEC	−0.198	−0.032	0.179	0.116	1						
	0.233	0.847	0.283	0.486							
Sand	.490**	−0.089	−0.249	0.087	−.430**	1					
	0.002	0.594	0.131	0.606	0.007						
Clay	−0.152	−0.076	0.115	−0.04	.842**	−.603**	1				
	0.363	0.649	0.493	0.811	0	0					
Soluble	−0.064	−0.134	0.198	0.088	−.349*	−0.07	−0.269	1			
	0.702	0.424	0.232	0.601	0.032	0.675	0.102				
Exchangeable	−0.319	−0.243	.353*	0.205	.552**	−.322*	.511**	0.064	1		
	0.051	0.142	0.03	0.217	0	0.048	0.001	0.703			
Non-exchangeable	−0.233	−0.019	0.293	0.211	.511**	−.496**	.540**	0.142	.796**	1	
	0.16	0.908	0.075	0.203	0.001	0.002	0	0.396	0		
Structural	−0.188	−0.114	0.295	0.081	.640**	−0.255	.630**	−0.276	.743**	.590**	1
	0.258	0.496	0.072	0.627	0	0.123	0	0.093	0	0	

** Correlation is significant at the 0.01 level (2-tailed).

* Correlation is significant at the 0.05 level (2-tailed).

### 3.3. Clay mineralogy

Clay mineral analysis (Table 3) identified illite, smectite, and chlorite as dominant, with minor quantities of vermiculite and kaolinite (<15%). The prevalence of illite was particularly evident, often decreasing with depth, except in pedon 10, where illite content increased—likely due to surface enrichment by wind-deposited materials or biotite transformation under fluctuating moisture–temperature cycles [12].

**Table 3:** Semi-quantitative analysis of soil clay content in the studied pedons.

Pedon	Horizon	Soil order	Smectite	Illite	Chlorite	Vermiculite	Kaolinite	Palygorskite
1	A	Entisols	**++**	**++**	**+++**	**+**	**+**	**–**
	C1		**++**	**++**	**+++**	**+**	**+**	**–**
2	Ap	Entisols	**++**	**+++**	**++**	**+**	**+**	**–**
	C1		**++**	**+++**	**++**	**+**	**+**	**–**
3	Ap	Entisols	**++**	**+++**	**++**	**+**	**+**	**–**
	C1		**++**	**+++**	**++**	**+**	**+**	**–**
4	Ap	Inceptisols	**+++**	**+++**	**++**	**+**	**+**	**–**
	Bw		**+++**	**++**	**++**	**+**	**+**	**–**
5	A	Entisols	**+++**	**+++**	**++**	**+**	**+**	**–**
	C1		**+++**	**++**	**++**	**+**	**+**	**–**
6	Ap	Inceptisols	**++**	**++++**	**++**	**+**	**+**	**–**
	Bw		**++**	**+++**	**++**	**+**	**+**	**–**
7	Ap	Vertisols	**++++**	**+++**	**++**	**+**	**+**	**–**
	Bss		**++++**	**++**	**++**	**+**	**+**	**–**
8	A_1_	Entisols	**++**	**+++**	**++**	**+**	**+**	**–**
	C1		**++**	**++**	**++**	**+**	**+**	**–**
9	A_1_	Aridisols	**++++**	**++**	**++**	**+**	**+**	**+**
	Bz		**++++**	**++**	**++**	**+**	**+**	**+**
10	Ap	Entisols	**+++**	**++**	**++**	**+**	**+**	**–**
	C		**+**	**+++**	**++**	**+**	**+**	**–**

Note: –, trace or not detected. + , content <15%; ++, content in the range of 15%–25%; +++, content in the range of 25%–35%; ++++, content 35%−45%.

Smectite dominated the surface horizons of pedons 7, 9, and 10, while illite prevailed in others. In the subsurface, pedons 5, 4, 7, and 9 showed a shift toward smectitic dominance. Vertisols (pedon 7) and Aridisols (pedon 9) contained the highest smectite percentages (35–45%). The presence of smectite in well-developed soils supports its pedogenic origin [13], although its formation may also result from transformation under high pH and Mg-rich environments. Smectite’s enrichment in subsurface horizons may reflect downward migration through eluviation processes [12]. Chlorite, found in substantial quantities across most pedons, showed irregular depth distribution. In pedon 1, chlorite was the dominant mineral at both depths, likely of inherited origin. Previous studies in southern Iran have attributed chlorite, illite, and kaolinite to lithological inheritance [12,13,25,45] . Palygorskite was observed only in pedon 9, characterized by high salinity and the presence of calcic–gypsic horizons. Palygorskite is often found in saline and alkaline conditions, where groundwater influences its formation. High concentrations of Mg²⁺ and silica in groundwater are crucial for palygorskite development [12,46].

### 3.4. Kinetics of K release

**Fig 3:**
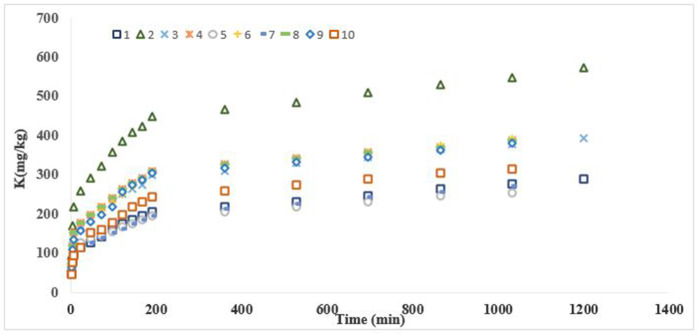
The trend of K release by 1 M CaCl_2_ for selected topsoils.

**Fig 4:**
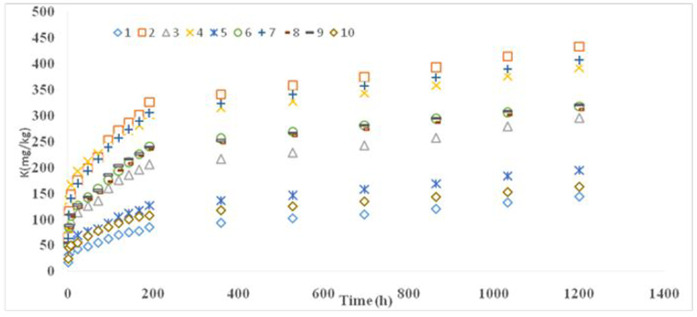
The trend of K release by 1 M CaCl_2_ for selected sub soils.

K release over time, studied via 0.01 M CaCl₂ extraction (Figs 3 and 4), followed a biphasic pattern: an initial rapid phase (2–192 min) attributed to surface site exchange, and a subsequent slower phase (192–1200 min) reflecting K release from interlayer and edge positions [47]. The stronger bonding of K within these sites, along with the larger hydrated radius of Ca² ⁺ relative to K ⁺ , slows ion exchange over time [20,48,49]. Modeling of K release data using pseudo-second-order, parabolic diffusion, Elovich, and power function equations revealed that the power function and pseudo-second-order models provided the best fit based on R² and SE values (Table 4) [20,50,51]. At the same time, the results were also fitted with zero and first-order equations, but the fitting results were poor. The b parameter of the power function was less than 1 in all cases, indicating that K release declined over time [52]. The highest a and b values were observed in pedons 5 (Entisols) and 6 (Inceptisols), respectively, with pedon 6 exhibiting. This pedon, containing 35–45% illite, 7.8% non-exchangeable K, 4.8% exchangeable K, and 1.12% soluble K, showed rapid and efficient K release, likely necessitating regular K fertilization to maintain soil fertility. According to Mengel and Uhlenbecker [53], the b parameter correlates with plant K uptake, emphasizing the importance of monitoring both exchangeable and non-exchangeable K. Without adequate K replenishment, especially in arid and semi-arid regions, soil K reserves may decline significantly over time [28]. Pseudo-second-order kinetics further confirmed these observations, with high R² values (0.9927 for surface and 0.9886 for subsurface soils) and low SE. The lowest rate constant (k = 0.0025) and highest equilibrium K (qe = 555.56) were recorded in pedon 2 (Entisols), suggesting a high K release potential but short equilibrium time. This profile had the lowest soluble and non-exchangeable K percentages, highlighting the importance of K fertilization during the growing season.

**Table 4:** Coefficient of determination (R^2^), standard error of the estimates (SE) and constants describing the kinetics of K release from soils.

	Power function					Pesudo-secend order			
	a	b	a*b	R^2^	SE	qe	K	R^2^	SE
Pedon	Surface horizons								
1	3.70	0.201	0.7437	0.90	0.138	285.71	0.0034	0.9899	0.0024
2	4.25	0.203	0.8627	0.87	0.169	555.56	0.0025	0.9950	0.0008
3	4.17	0.206	0.8590	0.90	0.015	384.62	0.0029	0.9936	0.0014
4	4.33	0.205	0.8876	0.88	0.168	400.00	0.0031	0.9944	0.0013
5	4.38	0.203	0.8891	0.87	0.168	263.16	0.0044	0.9912	0.0024
6	3.90	0.233	0.9087	0.85	0.202	400.00	0.0027	0.9929	0.0014
7	4.30	0.202	0.8686	0.85	0.162	277.78	0.0033	0.9888	0.0026
8	4.17	0.209	0.8715	0.85	0.183	400.00	0.0031	0.9944	0.0013
9	3.70	0.201	0.7437	0.90	0.132	400.00	0.0027	0.9930	0.0014
10	3.64	0.222	0.8081	0.86	0.204	333.33	0.0030	0.9937	0.0016
mean	4.054	0.2085	0.8443	0.873	0.154	374.94	0.0031	0.9927	0.0017
	a	b	a*b	R^2^	SE	qe	K	R^2^	SE
	Subsurface horizons								
1	3.61	0.192	0.6931	0.91	0.129	140.85	0.0045	0.9719	0.0081
2	3.98	0.206	0.8198	0.88	0.166	434.78	0.0025	0.9926	0.0014
3	3.69	0.214	0.7896	0.89	0.016	285.71	0.0033	0.9861	0.0028
4	3.99	0.208	0.8299	0.88	0.165	384.62	0.0034	0.9936	0.0014
5	4.19	0.201	0.8422	0.83	0.184	192.31	0.0040	0.9815	0.0048
6	3.70	0.229	0.8473	0.84	0.191	322.58	0.0033	0.9939	0.0017
7	3.86	0.204	0.7874	0.83	0.172	400.00	0.0028	0.9936	0.0013
8	3.73	0.225	0.8392	0.87	0.190	312.50	0.0034	0.9932	0.0018
9	3.97	0.211	0.8377	0.87	0.172	322.58	0.0033	0.9932	0.0017
10	3.16	0.227	0.7173	0.97	0.099	158.73	0.0054	0.9859	0.0050
mean	3.788	0.2117	0.8020	0.877	0.148	298.35	0.0036	0.9886	0.0030

## 4. Discussion

The relative abundance of clay minerals in the studied soils followed the general trend: illite > smectite > chlorite > vermiculite > kaolinite > palygorskite. Given the calcareous nature of the soils and the arid to semi-arid climate of the Darab region, most illite, chlorite, and kaolinite appear to be inherited from parent materials. In contrast, smectite and a portion of illite likely formed through pedogenic processes influenced by local climate, moisture availability, and soil development intensity. Pedon 9 (Aridisols), located in a lowland physiographic unit and characterized by 33.6% clay and CEC of 21.7 Cmol^(+)^/kg, exhibited the highest levels of exchangeable, non-exchangeable, and structural potassium. The dominance of smectite in this soil likely contributes to this observation, as smectite’s high surface area enhances K retention [54]. The presence of palygorskite in this pedon further supports its formation under saline conditions. Among the Entisols, pedon 8 had the highest percentage of soluble K, while pedon 3 contained the highest exchangeable and non-exchangeable K. These patterns can be attributed to higher clay content, greater CEC, and the dominance of illite. Illite is known to contribute to both exchangeable and non-exchangeable K pools and provides a buffering effect against K depletion [12,19]. In some contexts, its interaction with vermiculite may further enhance K availability, especially in humid conditions [8].

In most pedons, non-exchangeable K was more concentrated in the surface horizons compared to the subsurface, except in pedons 7, 9, and 10. This distribution suggests more intensive mineral weathering at the surface, driven by moisture and temperature fluctuations [55]. Weathering of micas and K-rich primary minerals likely contributes to the release of K into non-exchangeable forms at shallower depths [56]. On the other hand, structural K was generally more abundant in subsurface layers—further evidence of surface mineral breakdown and redistribution. A strong positive correlation was found between exchangeable K, CEC, and clay content, consistent with previous studies [57–61]. Since exchangeable K represents the portion held on mineral exchange sites, its availability depends largely on the type and amount of clay—particularly 2:1 and 2:1:1 clay minerals prevalent in these soils [62]. The inverse relationship observed between soluble K and CEC further supports the idea that stronger cation exchange capacity leads to greater K retention. K release experiments showed a two-phase kinetic pattern: a rapid initial release from external surfaces, followed by a slower, sustained release from interlayer and edge sites. This biphasic behavior reflects the binding strength of K in various mineral positions. Initially, K is readily exchanged with Ca²⁺ at external surfaces. Over time, the release slows due to the larger hydrated radius of Ca² ⁺ compared to K ⁺ , which impedes its ability to displace interlayer-bound K [57]. Interestingly, this slower exchange process may help maintain a more stable nutrient environment, supporting gradual plant uptake over time. Among the kinetic models tested, the power function and pseudo-second-order equations best fit the release data, as indicated by high R² and low SE values. The b parameter of the power function was consistently <1, indicating a declining release rate over time [52]. Pedon 6 (Inceptisols), which contained 35–45% illite and relatively high levels of non-exchangeable and exchangeable K, had the highest b suggesting efficient and rapid K release. Pseudo-second-order modeling yielded R² values of 0.9927 and 0.9886 for surface and subsurface soils, respectively. Pedon 2 (Entisols) showed the highest predicted K release capacity (qe = 555.56) and the lowest rate constant (k = 0.0025), indicating high K availability but a short time to equilibrium. These findings highlight the need for targeted K fertilization strategies, particularly during periods of high plant demand.

## 5. Conclusion

This study demonstrated that soil mineralogy, particularly the presence and distribution of illite and smectite, plays a crucial role in controlling potassium (K) dynamics in calcareous soils of the Darab region. Understanding how various forms of K behave across different soil types and depths provides valuable insight for improving nutrient management in arid and semi-arid environments.

- The dominant sequence of clay minerals across the studied soils was: illite > smectite > chlorite > vermiculite > kaolinite > palygorskite. While illite, chlorite, and kaolinite were mainly inherited from parent materials, smectite and some illite appeared to have both inherited and pedogenic origins.
- Among the studied pedons, pedon 9 (classified as an Aridisol), located in a lowland physiographic setting and containing 33.6% clay and a CEC of 21.7 Cmol(+)/kg, showed the highest concentrations of exchangeable, non-exchangeable, and structural K. Smectite was the dominant mineral in this profile, and the presence of palygorskite likely reflected the soil’s high salinity.
- The highest proportion of soluble K was recorded in pedon 8 (Entisol), whereas pedon 3 (also an Entisol) had the highest levels of exchangeable and non-exchangeable K. These findings are likely linked to higher clay content, elevated CEC, and the presence of illite as the dominant mineral.
- Non-exchangeable K was generally more concentrated in surface horizons, particularly due to enhanced weathering of primary and secondary minerals under surface temperature and moisture fluctuations. In contrast, structural K was typically higher in subsoil layers, providing further evidence of surface mineral transformation.
- Potassium release followed a two-phase kinetic pattern: an initial rapid release from external mineral surfaces, followed by a slower release from edges and interlayer positions. Over time, the exchange of K with calcium became less efficient due to the larger hydrated radius of Ca² ⁺ , leading to a gradual decline in the release rate.
- Release kinetics were best described by the power function and pseudo-second-order models. In all soils, the b parameter was less than one, indicating a decreasing release rate over time. Pedon 2 (Entisol) exhibited the highest K release potential, with the largest values for the a. Interestingly, this soil had the highest structural K content but the lowest non-exchangeable and soluble K, suggesting that a large reservoir of K was rapidly mobilized over a short time. The high qe and low k values further confirmed that K equilibrium was reached quickly in this profile.

In conclusion, the findings underscore the importance of considering both mineralogical composition and K release behavior when designing site-specific fertilization programs. Tailored management strategies are especially vital in dryland agricultural systems where maintaining long-term soil fertility is critical and nutrient losses can be difficult to recover.

### Deceleration of generative AI and AI-assisted technologies in the writing process

During the preparation of this work, the authors used (ChatGPT, Grok3, SCISPACE, etc.) for purpose of improving language, enhancing clarity and editing grammar. After using this tool/service, the authors reviewed and edited the content as needed and take full responsibility for the integrity and accuracy of the content of this publication.

## Supporting information

S1 File raw data-Supporting Information files. (XLSX)

## References

[1] Al-Silevany B, Mehmedany L. The Effect of Wetting and Drying Cycles on Potassium Release in Three Soil Orders. J Ecol Eng. 2023;24(1):218–26. doi: 10.12911/22998993/156055

[2] Elephant DE, Miles N, Muchaonyerwa P. Effect of Potassium Application Rates on Sugarcane Yield in Soils with Different Non-Exchangeable Potassium Reserves and Fixation Capacity. Agronomy. 2023;13(8):1969. doi: 10.3390/agronomy13081969

[3] A-Badrani W, Ali R. Status and distribution of different potassium forms in calcareous soils in northern Iraq. Mesopotamia Journal of Agriculture. 2019;47(2):178–91. doi: 10.33899/magrj.2019.163282

[4] Islam A, Saha PK, Biswas JC, Saleque MA. Potassium fertilization in intensive wetland rice system: yield, potassium use efficiency and soil potassium status. Int J Agric. 2016;1(2):7–21.

[5] Mengal K, Kirkby EA, Mengal K, Appel T, Kosegarton H. Principles of plant nutrition. Springer; 2006.

[6] Khormali F, Rezaei F, Rahimzadeh N, Hosseinifard SJ, Dordipour E. Rhizosphere-induced weathering of minerals in loess-derived soils of Golestan Province, Iran. Geoderma Regional. 2015;5:34–43. doi: 10.1016/j.geodrs.2015.02.002

[7] Affinnih K. Assessment of extractants for soil available potassium determination in the selected soils of Kwara state, Nigeria. Agricultural Sciences/Agrarni Nauki. 2023;15(36):39–45. doi: 10.22620/agrisci.2023.36.003

[8] Volf MR, Benites VM, Azevedo AC, Moraes MF, Tiritan CS, Rosolem CA. Soil mineralogy and K reserves in soils from the Araguaia River valley, Brazil. Geoderma Regional. 2023;33:e00654. doi: 10.1016/j.geodrs.2023.e00654

[9] Attia M. Status of Potassium in Some Calcareous Soils of Egypt and Factors Affecting Its Forms. Annals of Agricultural Science, Moshtohor. 2019;57(1):177–84. doi: 10.21608/assjm.2019.42247

[10] Barker AV, Pilbeam DJ. Handbook of plant nutrition. CRC press; 2015.

[11] Tan D, Liu Z, Jiang L, Luo J, Li J. Long-term potash application and wheat straw return reduced soil potassium fixation and affected crop yields in North China. Nutr Cycl Agroecosyst. 2017;108(2):121–33. doi: 10.1007/s10705-017-9843-0

[12] Shakeri S, Abtahi SA. Potassium forms in calcareous soils as affected by clay minerals and soil development in Kohgiluyeh and Boyer-Ahmad Province, Southwest Iran. J Arid Land. 2018;10(2):217–32. doi: 10.1007/s40333-018-0052-8

[13] Abolfazl Azadia, Sirous Shakeri. Potassium Pools Distribution in Some Calcareous Soils as Affected by Climatic Conditions, Physiographic Units, and Some Physicochemical Properties in Fars Province, Southern Iran. Eurasian Soil Sc. 2021;54(5):702–15. doi: 10.1134/s1064229321050021

[14] Islam S, Gathala MK, Timsina J, Dutta S, Salim M, Majumdar K. Potassium Supplying Capacity and Contribution of Non-Exchangeable Potassium in Wetland Rice Soils in Bangladesh. Communications in Soil Science and Plant Analysis. 2023;54(20):2745–62. doi: 10.1080/00103624.2023.2240853

[15] Enjavinezhad SM, Baghernezhad M, Abtahi SA, Ghasemi-Fasaei R, Zarei M. The effect of different classification levels and topography on potassium status of Sepidan soils, Fars province. Journal of Soil Management and Sustainable Production. 2023;13(1):75–93. doi: 10.22069/EJSMS.2023.20544.2071

[16] Shaikh K, Memon KS, Memon M, Akhtar MS. Changes in mineral composition and bioavailable potassium under long-term fertilizer use in cotton-wheat system. Soil Environ. 2007;26(1):1–9.

[17] Zareian G, Farpoor MH, Hejazi-mehrizi M, Jafari A. Kinetics of non-exchangeable potassium release in selected soil orders of southern Iran. Soil Water Res. 2018;13(4):200–7. doi: 10.17221/138/2017-swr

[18] Srinivasarao Ch, Rupa TR, Subba Rao A, Ramesh G, Bansal SK. Release Kinetics of Nonexchangeable Potassium by Different Extractants from Soils of Varying Mineralogy and Depth. Communications in Soil Science and Plant Analysis. 2006;37(3–4):473–91. doi: 10.1080/00103620500449351

[19] Zaarur S, Erel R. The effect of soil mineral composition on K availability to plants (No. EGU24-21838). Copernicus Meetings. 2024. 10.5194/egusphere-egu24-21838

[20] Jalali M. Kinetics of non-exchangeable potassium release and availability in some calcareous soils of western Iran. Geoderma. 2006;135:63–71. doi: 10.1016/j.geoderma.2005.11.006

[21] Bhat MA, Tomar D, Grewal KS, Sahoo J, Dar EA. Non-exchangeable potassium release and supplying power of soils of Haryana, Northwest India. Journal of Plant Nutrition. 2023;46(17):4140–55. doi: 10.1080/01904167.2023.2221683

[22] Islam A, Karim AJMS, Solaiman ARM, Islam MdS, Saleque MdA. Eight-year long potassium fertilization effects on quantity/intensity relationship of soil potassium under double rice cropping. Soil and Tillage Research. 2017;169:99–117. doi: 10.1016/j.still.2017.02.002

[23] Simonsson M, Andersson S, Andrist-Rangel Y, Hillier S, Mattsson L, Öborn I. Potassium release and fixation as a function of fertilizer application rate and soil parent material. Geoderma. 2007;140(1–2):188–98. doi: 10.1016/j.geoderma.2007.04.002

[24] Rosolem CA, Steiner F. Effects of soil texture and rates ofKinput on potassium balance in tropical soil. European J Soil Science. 2017;68(5):658–66. doi: 10.1111/ejss.12460

[25] Owliaie HR, Abtahi A, Heck RJ. Pedogenesis and clay mineralogical investigation of soils formed on gypsiferous and calcareous materials, on a transect, southwestern Iran. Geoderma. 2006;134(1–2):62–81. doi: 10.1016/j.geoderma.2005.08.015

[26] Barré P, Montagnier C, Chenu C, Abbadie L, Velde B. Clay minerals as a soil potassium reservoir: observation and quantification through X-ray diffraction. Plant Soil. 2007;302(1–2):213–20. doi: 10.1007/s11104-007-9471-6

[27] Sharma A, Sharma V, Arora S, Arya VM, Sankar GRM, Jalali VK. Potassium fixation capabilities of some inceptisols belonging to plain and sub-mountainous region. Jour of the Indian Socie of Soil Scie. 2016;64(4):368. doi: 10.5958/0974-0228.2016.00049.9

[28] Islam MJ, Cheng M, Kumar U, Maniruzzaman M, Nasreen SS, Haque ME, et al. Conservation agriculture in intensive rice cropping reverses soil potassium depletion. Nutr Cycl Agroecosyst. 2023;125(3):437–51. doi: 10.1007/s10705-023-10261-5

[29] Soil Survey Staff. Keys to Soil Taxonomy. 13th ed. USDA-Natural Resources Conservation Service; 2022.

[30] Bohn CC, Gebhardt K. Comparison of Hydrometer Settling Times in Soil Particle Size Analysis. Journal of Range Management. 1989;42(1):81. doi: 10.2307/3899665

[31] Nelson DW, Sommers LE. Total carbon, organic carbon, and organic matter. Methods of soil analysis: Part 2 chemical and microbiological properties. 1982. p. 539–79.

[32] Chapman H. Cation exchange capacity. Methods of soil analysis Part 2Chemical and microbiological properties. 1965. doi: 10.2134/agronmonogr9.2.c6

[33] Mehra OP, Jackson ML. Iron oxide removal from soils and clays by a dithionite–citrate system buffered with sodium bicarbonate. Clays and Clay Minerals. Elsevier. 2013. p. 317–27. doi: 10.1016/b978-0-08-009235-5.50026-7

[34] Kittrick JA, Hope EW. A procedure for the particle-size separation of soils for x-ray diffraction analysis. Soil Science. 1963;96(5):319–25. doi: 10.1097/00010694-196311000-00006

[35] Jackson ML. Soil chemical analysis-advanced course. 1969.

[36] Johns WD, Grim RE, Bradley WF. Quantitative estimations of clay minerals by diffraction methods. Journal of Sedimentary Research. 1954;24(4):242–51. doi: 10.1306/D42697B5-2B26-11D7-8648000102C1865D

[37] Mc Lean EO, Watson ME. Soil measurements of plant‐available potassium. Potassium in agriculture. 1985:277–308.

[38] Pratt PF. Potassium. Methods of Soil Analysis: Part 2 Chemical and Microbiological Properties. 1965:1022–30.

[39] Ghiri MN, Abtahi A, Jaberian F. Factors affecting potassium release in calcareous soils of southern Iran. Soil Res. 2011;49(6):529. doi: 10.1071/sr11098

[40] Hagin J, Feigenbaum S. Estimation of available potassium reserves in soils. 1962.

[41] Martin HW, Sparks DL. Kinetics of Nonexchangeable Potassium Release from Two Coastal Plain Soils. Soil Science Soc of Amer J. 1983;47(5):883–7. doi: 10.2136/sssaj1983.03615995004700050008x

[42] López-Piñeiro A, Navarro AG. Potassium release kinetics and availability in unfertilized vertisols of southwestern Spain. Soil Science. 1997;162(12):912–8. doi: 10.1097/00010694-199712000-00006

[43] Razmi B, Ghasemi-Fasaei R, Ronaghi A, Mostowfizadeh-Ghalamfarsa R. Application of Taguchi optimization for evaluating the capability of hydrochar, biochar, and activated carbon prepared from different wastes in multi-elements bioadsorption. Journal of Cleaner Production. 2022;347:131292. doi: 10.1016/j.jclepro.2022.131292

[44] Raheb A, Heidari A. Effects of clay mineralogy and physico-chemical properties on potassium availability under soil aquic conditions. J Soil Sci Plant Nutr. 2012;(ahead):0–0. doi: 10.4067/s0718-95162012005000029

[45] Najafi‐Ghiri M, Boostani HR. Effect of heating on some soil properties and potassium dynamics in calcareous soils of southern Iran. Soil Use and Management. 2020;37(3):519–32. doi: 10.1111/sum.12593

[46] Hojati S, Khademi H, Cano AF. Palygorskite Formation Under the Influence of Saline and Alkaline Groundwater in Central Iranian Soils. Soil Science. 2010;175(6):303–12. doi: 10.1097/ss.0b013e3181e0cbac

[47] Dhillon SK, Dhillon KS. Kinetics of release of non-exchangeable potassium by cation-saturated resins from Red (Alfisols), Black (Vertisols) and Alluvial (Inceptisols) soils of India. Geoderma. 1990;47(3–4):283–300. doi: 10.1016/0016-7061(90)90034-7

[48] Nascimento M, Da Cunha CD, Gonçalves F dos S. Potassium release behavior from silicate rocks using sulfuric and citric acids media. Rev DELOS. 2023;16(50):4257–73. doi: 10.55905/rdelosv16.n50-014

[49] Xue X, Zhang L, Peng Y, Li P, Yu J. Effects of Mineral Structure and Microenvironment on K Release from Potassium Aluminosilicate Minerals by Cenococcum geophilum fr. Geomicrobiology Journal. 2018;36(1):11–8. doi: 10.1080/01490451.2018.1485064

[50] Shakeri S, Abtahi A. Potassium fixation capacity of some highly calcareous soils as a function of clay minerals and alternately wetting-drying. Archives of Agronomy and Soil Science. 2019;66(4):445–57. doi: 10.1080/03650340.2019.1619176

[51] Cox AE, Joern BC, Brouder SM, Gao D. Plant‐Available Potassium Assessment with a Modified Sodium Tetraphenylboron Method. Soil Science Soc of Amer J. 1999;63(4):902–11. doi: 10.2136/sssaj1999.634902x

[52] Morad Wahba M. Evaluation of Kinetic Approach in Describing Potassium Bioavailability. AJHC. 2017;3(6):78. doi: 10.11648/j.ajhc.20170306.14

[53] Mengel K, Uhlenbecker K. Determination of Available Interlayer Potassium and Its Uptake by Ryegrass. Soil Science Society of America Journal. 1993;57(3):761–6. doi: 10.2136/sssaj1993.03615995005700030023x

[54] Nabiollahy K, Khormali F, Bazargan K, Ayoubi SH. Forms of K as a function of clay mineralogy and soil development. Clay miner. 2006;41(3):739–49. doi: 10.1180/0009855064130216

[55] Wakeel A, Ishfaq M, Wakeel A, Ishfaq M. Potassium dynamics in soils. Potash Use and Dynamics in Agriculture. 2022:7–17. doi: 10.1007/978-981-16-6883-8_2

[56] Kaur H. Forms of Potassium in Soil and their Relationship with Soil Properties- A Review. IntJCurrMicrobiolAppSci. 2019;8(10):1580–6. doi: 10.20546/ijcmas.2019.810.184

[57] Chand S, Swami BN. Different forms of potassium in some important soil associations of Bharatpur district of Rajasthan. J Potassium Res. 2000;16:59–1. https://doi/full/10.5555/20013132265

[58] Das K, Sarkar D, Nayak DC. Forms of potassium and their distribution in some soils representing red and laterite ecosystem of West Bengal. J Potassium Res. 2000;16:1–6.

[59] Gangopadhyay SK, Sarkar D, Sahoo AK, Das K. Forms and distribution of potassium in some soils of Ranchi plateau. J Indian Soc Soil Sci. 2005;53:413–6.

[60] Kundu MC, Hazra GC, Biswas PK, Mondal S, Ghosh GK. Forms and distribution of potassium in some soils of Hooghly district of West Bengal. J Crop Weed. 2014;10(2):31–7.

[61] Singh K, Malik RVS, Sing V. Distribution of forms of potassium in alluvial soils. J Potassium Res. 2001;17:116–8.

[62] Sposito G. The chemistry of soils. Oxford University Press. 2008.

